# Dual Anticoagulation in Recurrent Thromboembolic Events with Failure of Monotherapy: A Novel Approach

**DOI:** 10.7759/cureus.1444

**Published:** 2017-07-07

**Authors:** Lakshmi Manogna Chintalacheruvu, Osman Bhatty, Venakata giri Andukuri

**Affiliations:** 1 Internal Medicine, Creighton University School of Medicine

**Keywords:** venous thromboembolism, dual anticoagulant therapy

## Abstract

In the last few decades many new anticoagulants (i.e direct thrombin and factor ten inhibitors) have been introduced with efficacy that rivals older drugs in the treatment of venous thromboembolism (VTE). However, for all their success, management of patients with recurrent thromboembolic events is still a challenging clinical scenario and not well addressed in the literature. We report the case of a young female with recurrent thromboembolisms in spite of using both newer agents and more conventional therapies. Ultimately, she is started on dual anticoagulation with warfarin and rivaroxaban without recurrence. This case report demonstrates that dual anticoagulants can be utilized in patients with recurrent VTE who fail single agent therapy. It also underscores the need for studies further elaborating on the utility of dual anticoagulants as a treatment modality for patients failing monotherapy.

## Introduction

Venous thromboembolism (VTE) includes both venous thrombosis and pulmonary embolism (PE). It is associated with significant morbidity and mortality with a 30-day death rate of about 3% and 31%, respectively, in those left untreated [1]. Despite being on therapeutic doses of anticoagulation, patients can still develop recurrent PE, which is appropriately termed “anticoagulation failure.” The rate of recurrent PE is up to 4% with low-molecular-weight heparin (LMWH) and 2-4% with vitamin K antagonists (VKA) [1].

## Case presentation

A 32-year-old Caucasian female presented to the emergency department with an acute onset of shortness of breath (SOB). Her past medical history was significant for recurrent VTE of unknown etiology with removal of an inferior vena cava filter due to misplacement. She had no family history of thromboembolic disorder and no past medical history of smoking or oral contraceptive usage. Her first episode of PE was spontaneous about six years ago followed by multiple episodes of VTE that required thrombolysis on three separate occasions. Her comprehensive hypercoagulable workup in the past included factor V Leiden mutation, JAK2 V617 mutation, Lupus anticoagulant, antithrombin III activity, PNH flow cytometry, factor II gene mutation, protein C, protein S, anti-cardiolipin antibody, anti-beta-2 glycoprotein-1 antibody, and homocysteine levels, which were all unremarkable. She had recurrent PE on many therapies including warfarin (with therapeutic international normalized ratio of 2.5-3.5), rivaroxaban, apixaban, dabigatran, heparin, and fondaparinux. She also had a history of rash secondary to enoxaparin making management more complicated. Her pertinent physical exam findings on presentation included hypoxia on 2 liters of nasal cannula with oxygen saturation at 96%, respiratory rate of 22 and decreased breath sounds bilaterally in the lung bases with no signs of deep vein thrombosis (DVT). A computed tomography (CT) scan of the chest with contrast on admission showed new pulmonary emboli on the right side.

At this point it was challenging to decide the next step in management since she had failed most known anticoagulants in the past. This also caused much physical, psychological, and financial burden on the patient due to recurrent hospitalizations over a short period of time.

The hematology service was consulted, and after a thorough discussion with the patient, rivaroxaban 15 mg twice daily was initiated since she had failed the 20 mg once daily dose in the past. Unfortunately, after about three weeks of rivaroxaban treatment she presented with another episode of PE. She was started on therapeutic heparin at this time with activated partial thromboplastin time (aPTT) range between 90-140 seconds. A higher aPTT range was selected given her recurrence. She required large doses of heparin to maintain her aPTT, but after it was maintained at the new therapeutic goal, her breathing eventually improved and she did not require further oxygen supplementation. At this time, the hematology service decided to combine two oral anticoagulants to prevent further episodes of PE since she has had recurrence on heparin. They initially recommended combining a second oral anticoagulant with rivaroxaban such as apixaban or dabigatran, but due to the patient’s health insurance coverage issues and the higher price of novel oral anticoagulants, two drugs from this class did not seem feasible. Instead, she was started on warfarin with a therapeutic goal INR range of 2-3 along with rivaroxaban 15 mg bis in die (BID). At the time of writing this manuscript, six months since the patient was seen, she has had no recurrence of PE or signs of bleeding.

## Discussion

Venous thromboembolism (VTE), including DVT of the extremities or pelvis and PE, is associated with a significant morbidity and mortality with approximately 60,000 to 100,000 deaths in the United States every year [2]. Anticoagulation is the mainstay of treatment in patients with VTE, and aggressive management is essential in decreasing mortality [2].

The optimal management of anticoagulation failure includes dose escalation, switching to a different anticoagulant or adding an antiplatelet drug [3]. The literature was reviewed to find the utility of adding an antiplatelet drug such as aspirin in our patient, but it was shown to only be beneficial in antiphospholipid syndrome [3] and not in other patient groups. We trialled all the aforementioned strategies before ultimately placing the patient on dual anticoagulants including dose escalation of rivaroxaban and using different anticoagulants. Unfortunately, she had recurrence of thromboembolic events in each case.

Another approach to anticoagulation failure is by combining anticoagulants [2]. Two case reports in the literature were found pertaining to dual anticoagulation [2]. The first case report included a 43-year-old female with clear adenocarcinoma of the ovary who presented with recurrent VTE despite being on a therapeutic dose of dalteparin [2]. Coumadin was added to dalteparin with a therapeutic INR goal of 2-3, after which there was no recurrence of thromboembolic events [2]. The second case report was a 57-year-old male with colon adenocarcinoma who was started on dual anticoagulation with warfarin and dalteparin after he failed dalteparin monotherapy [2]. In both case reports, thromboembolic events were prevented with no bleeding episodes for more than three to four months [2]. Of note, the patients in these case reports had a diagnosis of cancer, and the utility of dual anticoagulant therapy in patients without a diagnosis of cancer is not well established.

We did an extensive literature review and could not find any case reports utilizing dual anticoagulant therapy in the management of VTE in those without a diagnosis of cancer [2]. There is no evidence to our knowledge to support the practice of combining warfarin with other oral anticoagulants, which was ultimately done successfully in our patient since she failed a large variety of monotherapies. The only evidence we found at this time towards dual anticoagulants is combining LMWH with warfarin. Unfortunately, this could not be done with our patient since she had developed a rash with enoxaparin in the past [2].

## Conclusions

In summary, this case report is unique in demonstrating that dual anticoagulation can be used in patients with recurrent VTE especially when monotherapies have failed them. Caution should be exercised in evaluating the benefits and risks (mainly bleeding) before giving this treatment. Further studies are needed to determine the optimal combination of anticoagulation with minimal bleeding risk.

## References

[1] Galioto NJ, Danley DL, Van Maanen RJ (2017). Recurrent venous thromboembolism. Am Fam Physician.

[2] Pillai AR, Olujohungbe A, Evans MR (2010). The management of recurrent VTE in cancer patients receiving therapeutic anticoagulation: the use of dual anticoagulant therapy combined with an IVC filter. Blood Coagul Fibrinolysis.

[3] Kazmi RS, Lwaleed BA (2011). New anticoagulants: how to deal with treatment failure and bleeding complications. Br J Clin Pharmacol.

